# Plant Phylogeny and Life History Shape Rhizosphere Bacterial Microbiome of Summer Annuals in an Agricultural Field

**DOI:** 10.3389/fmicb.2017.02414

**Published:** 2017-12-11

**Authors:** Bryan D. Emmett, Nicholas D. Youngblut, Daniel H. Buckley, Laurie E. Drinkwater

**Affiliations:** ^1^Horticulture Section, School of Integrative Plant Science, Cornell University, Ithaca, NY, United States; ^2^Department of Microbiome Science, Max Planck Institute for Developmental Biology, Tübingen, Germany; ^3^Soil and Crop Sciences Section, School of Integrative Plant Science, Cornell University, Ithaca, NY, United States

**Keywords:** exoenzymes, microbiome, nitrogen use efficiency, plant phylogeny, rhizosphere

## Abstract

Rhizosphere microbial communities are critically important for soil nitrogen cycling and plant productivity. There is evidence that plant species and genotypes select distinct rhizosphere communities, however, knowledge of the drivers and extent of this variation remains limited. We grew 11 annual species and 11 maize (*Zea mays* subsp. *mays*) inbred lines in a common garden experiment to assess the influence of host phylogeny, growth, and nitrogen metabolism on rhizosphere communities. Growth characteristics, bacterial community composition and potential activity of extracellular enzymes were assayed at time of flowering, when plant nitrogen demand is maximal. Bacterial community composition varied significantly between different plant species and genotypes. Rhizosphere beta-diversity was positively correlated with phylogenetic distance between plant species, but not genetic distance within a plant species. In particular, life history traits associated with plant resource acquisition (e.g., longer lifespan, high nitrogen use efficiency, and larger seed size) were correlated with variation in bacterial community composition and enzyme activity. These results indicate that plant evolutionary history and life history strategy influence rhizosphere bacterial community composition and activity. Thus, incorporating phylogenetic or functional diversity into crop rotations may be a tool to manipulate plant-microbe interactions in agricultural systems.

## Introduction

The rhizosphere is a hotspot of plant-microbe interactions with profound influence on plant productivity and ecosystem function (Philippot et al., 2013). Shaped by the release of labile carbon (C) from plant roots and root uptake of nutrients and water (Hinsinger et al., 2005), the physiochemical environment of the rhizosphere supports a microbial community compositionally and metabolically distinct from that found in bulk soil (Mendes et al., 2014). The resulting rhizosphere microbiome performs critical functions, modulating plant growth and development (Panke-Buisse et al., 2015), plant health (Mendes et al., 2011; Berendsen et al., 2012), and plant nutrient acquisition (Philippot et al., 2013; Pii et al., 2015).

Nitrogen (N) is a limiting nutrient in most terrestrial ecosystems and plant–microbe interactions in the rhizosphere govern many N transformations in soil. The C-rich and N-limited environment of the rhizosphere is a site of associative N-fixation (James, 2000), and also frequently a site of increased decomposition and subsequent N mineralization of soil organic N pools (Kuzyakov, 2002; Herman et al., 2006). While the details regulating this “rhizosphere priming effect” are still poorly understood, it is broadly thought improved C status of the rhizosphere relieves energetic constraints on microbial activity and production of extracellular enzymes that breakdown soil organic matter (Averill and Finzi, 2011; Dijkstra et al., 2013). The activity of these enzymes is a rate limiting step in decomposition and subsequent N mineralization (Schimel and Bennett, 2004) and increased rates of N cycling that follow can feed back and support plant N acquisition (Hamilton and Frank, 2001; Zhu et al., 2014), particularly when coupled with the turnover or predation of microbial populations (Clarholm, 1985).

The importance of plant-microbial collaborations in plant nutrient acquisition presents an opportunity to modify crop-breeding approaches to select genotypes that foster rhizosphere microbiomes that can decrease the need for surplus additions of N fertilizer (Drinkwater and Snapp, 2007; Wissuwa et al., 2009). Therefore there is considerable interest in understanding the factors governing the assembly and function of the rhizosphere microbiome. An emerging picture suggests soil background is a dominant force in shaping bacterial community composition (BCC) in the rhizosphere. Within a soil context, plant species and genotypes influence this community (Berg and Smalla, 2009; Peiffer et al., 2013; Bulgarelli et al., 2015; Edwards et al., 2015), which can in turn be modulated by plant developmental stage and plant health status (Zhang et al., 2011; Chaparro et al., 2014; Marques et al., 2014). However, while some studies observe strong plant identity effects, others report no or limited effects (Wagner et al., 2016; Leff et al., 2017), and therefore understanding the sources and extent of plant-driven variation in the composition and function of the rhizosphere bacterial community remains a critical research challenge.

Presumably, variation in rhizosphere community composition and function is most likely driven by the evolutionary and ecological differentiation of host plants. For instance, several host–microbe interactions display a phylogenetic signal, such that closely related species share more similar microbiomes than distantly related species (Ley et al., 2008; Brucker and Bordenstein, 2012b). Such a phylogenetic signal has been observed in the rhizosphere of the Poaceae (Bouffaud et al., 2014), and in the phyllosphere of a broad range of plants, where increasing beta-diversity can be observed at the species, order and division levels (Redford et al., 2010). These patterns can arise from either specific co-evolutionary processes (Brucker and Bordenstein, 2012a) or, as proposed by Bouffaud et al. (2014), from microbiome assembly driven by the ability of phylogenetically conserved plant traits to shape microbial niche space in the rhizosphere.

Conversely, ecological differentiation among closely related hosts may interact with such a phylogenetic signal. For instance, diet is a significant driver of the mammalian gut microbiome and only after controlling for diet is a phylogenetic relationship between mammalian hosts and microbiome composition evident (Ley et al., 2008). Plant uptake of N and release of C are among several factors that shape the rhizosphere physiochemical environment (Hinsinger et al., 2005; Bell et al., 2015), therefore plant traits governing N and C acquisition and use may be strongly linked with plant variation in rhizosphere composition (Zancarini et al., 2013).

Plants adapt to varying levels of N availability through their competitive ability to acquire N from soil and their nitrogen use efficiency (NUE) defined broadly as the amount of C fixed per unit plant N (Vitousek, 1982). Both strategies, N-acquisition and NUE, may affect rhizosphere communities. The rate of plant N uptake likely shapes plant-microbe competition for N. Correspondingly, differences in rhizosphere BCC have been observed between genotypes or plant species that differ in rates of N uptake (Moreau et al., 2015; Pathan et al., 2015), and these differences extend to indicators of N-cycling and extracellular enzyme activity (Cantarel et al., 2015; Pathan et al., 2015). Conversely, NUE is often associated with improved N retention in plant tissues (Berendse and Aerts, 1987), and plant traits promoting tissue longevity and N retention (e.g., increased tissue thickness, lignin content and decreased N content) are associated with decreased rates of decomposition and nutrient-cycling in soils under high NUE plants (Diaz et al., 2004; Orwin et al., 2010). These nutrient-cycling effects may be an indirect consequence of variation in litter quality, but it is also possible that these effects are mediated by direct plant impacts on microbiome composition and function.

To investigate the sources and extent of plant variation in rhizosphere effects, we conducted a common garden experiment with a selection of maize inbred lines and summer annual species commonly found in agricultural systems. We characterized BCC and enzyme activity in plant rhizospheres to test hypotheses that (1) plant rhizosphere effects vary according to the evolutionary history of host species, and (2) that variation in rhizosphere BCC and metabolism is associated with variation in plant growth characteristics and nitrogen economy.

## Materials and Methods

### Experimental Design

A common garden experiment was conducted at the Musgrave Research Station in Aurora, NY (42°44′11′′N 76°39′05′′W). The soil at the site is classified as fine-loamy, mesic Oxyaquic Hapludalfs, with a circumneutral pH of 7.65 and consisted of 45.2% sand, 33.5% silt and 21.3% clay. The soil was 1.7 ± 0.14% carbon and 0.17 ± 0.14% N; inorganic N (NH_4_ + NO_3_) content at tillage was 7.1 ± 1.3 μg g^-1^. Mehlich extractable P and K concentrations were 19.5 ± 1.5 μg g^-1^ and 146 ± 16 μg g^-1^, respectively. The field was previously managed as a corn-soy rotation and had been planted to corn in the previous year. Prior to planting the field was moldboard plowed, disked, fit for planting and fertilized with 224 kg ha^-1^ of potassium phosphate (0-15-30).

Plants were selected to encompass a range of intra- and interspecific diversity found in agricultural fields. This included ten founding inbred lines of the maize (*Zea mays* subsp *mays* L.) Nested Association Mapping (NAM) population, which represents the genetic diversity of improved maize (Yu et al., 2008), as described by Peiffer et al. (2013). Lines were chosen to represent differences in growth, N uptake and yield under fertilized and unfertilized conditions (Meyer, 2006). Additionally, one inbred line (75-062) was included from a public organic breeding program. We broadened phylogenetic and functional variation by including eight C4 grasses [*Echinochloa crus-galli* (L.) P.Beauv, *Setaria faberi* R.A.W.Herrm., *Eragrostis tef* (Zucc.) Trotter, *Sorghum bicolor* (L.) Moench subsp. *bicolor, Sorghum* X *drummondii* (Nees ex Steud.) Millsp. & Chase, and *Eleusine coracana* (L.) Gaertn.], four dicots (*Abutilon theophrasti* Medik., *Amaranthus powellii* S.Watson, *Helianthus annuus* L., *Fagopyrum esculentum* Moench), and a legume [*Glycine max* (L.) Merr.] (Supplementary Table S1).

Replicated monocultures were planted on June 19th and 21st, 2013 in a split-plot randomized complete block design (*n* = 4). Plots consisted of eight 1.83 m rows spaced at 76 cm, with 23 cm between plants in a row, resulting in a final density of 57,500 plants ha^-1^. Each main plot was split such that half the rows received a nitrogen application of 23.5 kg N ha^-1^ at planting and two side-dress applications (July 11th and August 5th) totaling 95 kg N ha^-1^ as (NH_4_)_2_SO_4_, while the remaining rows received no N fertilizer. This fertilizer level was chosen to boost plant growth but not provide luxury N conditions. Granular side-dress N was hand applied throughout the plot and incorporated during cultivation. Plots were kept weed free through mechanical cultivation and hand weeding.

### Plant and Rhizosphere Sampling

Plants were harvested when at least 50% of the flowers/tassels for that genotype were shedding pollen. Since the phenology of these species are not synchronized this resulted in eight harvests (Supplementary Table S1). By sampling at a common developmental stage we control for the effects of plant developmental stage on rhizosphere BCC (Chaparro et al., 2014; Marques et al., 2014) and by sampling at anthesis, when plant biomass accumulation and nutrient uptake are maximal, we are able to evaluate rhizosphere composition when it is most relevant for nutrient uptake of each species. Three to four adjacent and representative plants from an interior row of each plot were clipped at the first nodal roots and dried at 60°C for dry weight determination. Homogenized and ground tissue was analyzed for tissue C and N content on a PDZ Europa ANCA-GSL elemental analyzer at the University of California Davis Stable Isotope Facility.

At sampling, root systems were loosened from the ground with a spade and soil loosely adhered to the root system was removed by massaging and gentle shaking and discarded. Soil that remained adhered to the roots was considered rhizosphere soil and gently removed with a gloved hand, passed through a 2 mm sieve, and bagged for downstream analysis of inorganic N content and potential extracellular enzyme activity. Additionally, intact roots with adhering rhizospheres were sampled by clipping randomly selected 4 cm segments of root tips and parent 2nd order roots for nucleic acid analysis. On each sampling date, 2 cm diameter by 20 cm deep soil cores were collected from unplanted, weed-free plots to represent bulk/bare soil in downstream analyses. Multiple cores were combined, homogenized, subsampled and passed through a 2 mm sieve. All samples were immediately placed on ice and then stored at 4°C for downstream analysis of enzymes and inorganic N content and at -40°C for nucleic acid analysis.

### Extracellular Enzyme Analysis

Potential activity of enzymes involved in degradation of hemi-cellulose [β-xylosidase (BX)], cellulose [cellobiohydrolase (CB)], protein [leucine aminopeptidase (LAP)] and chitin [β-*N*-acetyl-glucosaminidase (NAG)] were measured using standard fluorometric assays following German et al. (2011). Briefly, 2–3 g field moist soil was mixed with 150 ml of 50 mM sodium bicarbonate buffer adjusted to pH 8 for 60 s using an immersion blender. 200 μl of soil slurry was added to 8 replicate wells of a 96-well plate containing 50 μl of 200 μM substrate with attached fluorophore. LAP plates were incubated for 2 h and BX, CB, and NAG incubated for 4 h at 30°C. Fluorescence was measured on a BioTek Synergy HT microplate reader at 365 nm excitation and 450 nm emission. Enzyme activity for each soil was estimated using a standard curve (0–75 μM) prepared from the same homogenate to control for quenching and autofluorescence. Standard curves were made fresh daily. To fix a perceived degradation of our standard over the season, the curves from each date were scaled so the maximum florescence of the 50 μM standard was equal across dates. All enzyme analyses were completed within 48 hrs of sample collection. Subsamples of soil were dried at 60°C for 48 h for soil moisture determination. Enzyme activity is expressed on a soil dry weight basis (nmol g soil^-1^ hr^-1^).

### Inorganic Nitrogen Determination

A subset of species and genotypes were chosen to collect sufficient rhizosphere soil for inorganic N determination (Supplementary Table S2). Inorganic nitrogen was extracted in duplicate from 8 to 10 g of rhizosphere or bulk soil in 40 ml of 2 M KCl, shaken for 1 h and filtered through pre-rinsed ashless Whatman filter paper. Extracts were analyzed colorimetrically for nitrate and ammonium concentration using a VCl_3_/Griess method (Miranda et al., 2001) and modified indophenol method (Kandeler and Gerber, 1988), respectively, in a 96-well microplate format following Doane and Horwáth (2003) and Hood-Nowotny et al. (2010). Plates were incubated at 37°C for 2 h for nitrate determination and 30 min at 21°C for ammonium determination. Absorbance of wells was analyzed on a BioTek Synergy HT microplate reader at 540 and 660 nm, respectively. Concentrations were calculated using a standard curve included on each plate and expressed on a soil dry weight basis (μg N g soil^-1^).

### 16S rRNA Gene Sequence Analysis

Root and rhizosphere and bare soil samples stored at -40°C were lyophilized for 24 h on a LabConco FreeZone 2.5 freeze dry system. Roots were chopped to <1 cm length segments, mixed, and between 0.01 and 0.05 g of freeze dried roots and adhering soil or 0.15 g of bare soil controls were added directly to each well of a 96-well extraction plate from the MoBio PowerSoil-htp DNA kit (Carlsbad, CA, United States). Rhizosphere samples were added to duplicate wells to adequately capture heterogeneity of the root systems. Samples were homogenized on a BioSpec Mini-Beadbeater-96 (Bartlesville, OK, United States) for 2 min and extractions proceeded according to manufacturer’s instructions. The 0.7 mm bead size of the PowerSoil kit does not homogenize root tissue and roots remained largely intact following homogenization (Peiffer et al., 2013). However, there was likely disruption of epidermal and some cortical cells and it is likely that our extracts contained some DNA from root endophytes. Following bead beating, extraction proceeded according to the kit manufacturer’s instructions. DNA yields were quantified with the Quant-iT PicoGreen dsDNA Assay Kit (Invitrogen). Extractions yielded a mean of 375 ± 224 ng DNA template for use in downstream applications.

Dual-barcoded MiSeq libraries of the SSU rRNA V4 region were prepared as in Kozich et al. (2013) using the forward (515F) (Whitman et al., 2016) and reverse primers (806R) adapted from Caporaso et al. (2010). Amplicons were prepared in triplicate reactions. Each reaction included 5 ng of template DNA, 12.5 μl of 2x Q5 High Fidelity, Hot Start PCR Mastermix, 1 μM combined forward and reverse primer, 0.5 μg bovine serum albumin and 0.625 μl of 4x PicoGreen reagent to monitor DNA template production for a total volume of 25 μl. PCR conditions consisted of: 95°C for 2 min; 30 cycles of 95°C for 20 s, 55°C for 15 s and 72°C for 10 s; final extension 72°C for 5 min. Pooled triplicate reactions were standardized using the SequalPrep Normalization Plate Kit (Life Technologies). Standardized reactions were pooled then gel purified and extracted using the Wizard SV Gel and PCR Clean-Up System (Promega). The two resulting amplicon libraries were submitted for 2 × 250 bp paired-end sequencing on the Illumina MiSeq platform with the MiSeq Reagent v2 kit at the Cornell Biotechnology Resource Center Genomics Facility (Ithaca, NY, United States).

Resulting reads were processed in a custom bioinformatics pipeline as in Whitman et al. (2016). Overlapping paired-end reads were merged using PEAR (v0.9.2) (Zhang et al., 2014). Merged reads were de-multiplexed with a custom python script and those that did not match a known barcode were discarded. Remaining reads were filtered to remove sequences with max expected error rates > 1 with USEARCH (Edgar, 2013), ambiguous base calls, ≥8 homopolymers and singletons (unique). Sequences were clustered into operational taxonomic units (OTUs) at a 97% pairwise identity cutoff with USEARCH (Edgar, 2013). Taxonomic assignment of OTUs was performed with Qiime’s parallel taxonomy assignment using the uclust consensus taxonomy assignment function (Caporaso et al., 2010) and the Silva reference database (v.111) (Quast et al., 2013). OTUs belonging to chloroplast, mitochondria, eukaryotes, archaea and unassigned sequences were removed. OTUs were aligned using SSU_align and poorly aligned positions masked based on posterior probabilities (Nawrocki, 2009). A phylogenetic tree was created and rooted to *Sulfolobus* (acc. X90478) using FastTree (Price et al., 2009) with default settings. The resulting OTU table contained 11,246 OTUs representing 7,517,735 mapped reads and was combined with the phylogenetic tree, taxonomic information and metadata for analysis using the phyloseq package in R (McMurdie and Holmes, 2013). This OTU table was further filtered using a sparsity threshold of greater than three reads in more than three samples in order to remove extremely rare taxa, but retain taxa that may be endemic in the rhizosphere of a particular genotype, which resulted in 4982 OTUs. Sequences and associated metadata were deposited in the NCBI sequence read archive under accession #SRP119673.

### Phylogeny and Genetic Distance Matrices

Chloroplast rbcL and matK genes were used to construct a phylogeny of the twelve plant species. Representative sequences were downloaded from the GenBank Nucleotide Database. *Amaranthus powellii* was not represented and sequences from congeneric *A. viridis* were used instead. Sequences were aligned and checked in Unipro UGENE (Okonechnikov et al., 2012) and a tree was constructed using phyloGenerator with *Ginkgo biloba* used as an outgroup (Pearse and Purvis, 2013). A distance matrix was derived using the cophenetic.phylo function in the R package “ape” (Paradis et al., 2004). We expected little intraspecific variation in chloroplast rbcL or matK genes and arbitrarily assigned a distance of 0.0002 to intraspecific comparisons. This approximates intraspecific distances among maize lines found in previous studies using chloroplast markers (Bouffaud et al., 2014). Genetic distance matrices for the ten NAM inbred lines were constructed using GBS markers build 2.7 available at panzea.org. Distance matrices were estimated using TASSEL version 5 (Bradbury et al., 2007).

### Statistical Analysis

Statistical analyses were conducted in R (R Development Core Team, 2012). To compare growth of plants sampled on different days, log-transformed plant biomass, N uptake and NUE (g C g N^-1^) were modeled by days after planting (DAP) (Supplementary Figure S1) for all species with one representative of maize (cv. B73). Data from an early season biomass cut was included to improve the model of plant growth over the course of the season (Supplementary Figure S1). Inbred maize lines were modeled separately in order to avoid weighting the model of plant growth. Residuals from the best-fit line were used to estimate variation in plant growth characteristics independent of flowering time.

Univariate tests were conducted in the package “lme4” (Bates et al., 2015) and *p*-values estimated with “lmerTest” (Kuznetsova et al., 2016). Sample type, plant genotype, fertilization and interactions were considered fixed effects with the random effects of replicate block and split-N fertilization plots. A similar mixed model was used to test the influence of rhizosphere inorganic N concentration on potential extracellular enzyme activity. Here, plant genotype was included as a random effect to control for influence of plant genotype and date of sampling on enzyme activity. *Post hoc* tests were conducted using the glht function in the “multcomp” package (Hothorn et al., 2008).

Bacterial community beta-diversity was analyzed using weighted-UniFrac distance matrices (Lozupone et al., 2011) constructed using an OTU table rarified to 4989 reads per sample in the phyloseq package in R (McMurdie and Holmes, 2013). Treatment effects on beta-diversity were tested using permutational multiple analysis of variance (PERMANOVA) using the “adonis” function in the Vegan package (Oksanen et al., 2012). We tested the effect of phylogenetic distance, maize whole genome genetic distance, and variation in growth characteristics between plant hosts on rhizosphere BCC using a generalized least squares implementation of Clarke’s maximum likelihood population effects model (MLPE) (Clarke et al., 2002), using the R function corMLPE^1^. The MLPE allows correlation between distance matrices by using a random effect parameter to estimate residual covariance of observations sharing a common sample, which would otherwise violate the assumption of independent observations (Clarke et al., 2002). To avoid pseudo-replication in the analysis of phylogenetic and genetic distance, the weighted-UniFrac distance matrix was calculated on OTU tables averaged over each genotype. In the models of phylogenetic and genetic distance, the sampling dates of each pairwise plant comparison was included as a fixed effect to control for variation between sampling dates. These models were evaluated using a likelihood ratio test against the nested null model of sampling date. Analyses of interspecific variation were conducted using all species with one representative of maize (cv. B73), while intraspecific analyses were conducted using the maize inbred lines. When not explicitly stated, analyses were conducted using all samples. To further explore the role of plant growth characteristics in shaping interspecific variation in rhizosphere BCC we constrained the principle coordinate analysis of weighted-UniFrac distances to display only variation that could be explained by plant growth metrics, using the “CAP” method of the “ordinate” function in phyloseq.

The response of individual OTUs to treatments and correlation with covariates was calculated as log_2_-fold change using non-rarified OTU table in a negative binomial model within the DESeq2 package (Love et al., 2014) and Benjamini and Hochberg corrected *p*-values reported. Rhizosphere responders were identified as those OTUs with a significant positive log_2_-fold change greater than 0.5 between a genotype’s rhizosphere and the bare soil controls sampled on the same date. When testing the role of rhizosphere inorganic N concentration or fertilization on OTU abundance plant genotype was included in the model to control for variation between plants and between sampling dates. All figures were created in the package “ggplot2” (Wickham, 2009) except the circular phylogenetic tree, which was created using the interactive tree of life (iTOL) web server (Letunic and Bork, 2016). Final annotation and formatting of figures was performed in Inkscape. Scripts for bioinformatics pipeline, analysis and figure generation are available at https://github.com/bdemmett/RhizCG.

## Results

### Variation in Plant Growth and Nitrogen Economy

Across plant species and genotypes we observed nearly 10-fold variation in biomass accumulation and N uptake, and 4-fold variation in NUE at anthesis (Supplementary Figure S2). This variation derived primarily from differences in flowering time and sampling date. Flowering time ranged from 36 DAP for *F. esculentum* to 88 DAP for *E. coracana*, and from 72 to 88 DAP for short and long season maize lines (Supplementary Table S1). As a result, DAP captured 69 and 77% of the variation in log-transformed N uptake and NUE among the plant species sampled (91 and 86% among maize inbred lines, respectively) (Supplementary Figure S1). While this is expected, as longer-lived plants have more time to grow and acquire N from soil, it highlights the variation in resource demand among annual plants. The longer-lived plants had both greater N demand and greater NUE owing to increased effective retention time.

Residuals from the models above were used to evaluate differences in plant growth and N economy, independent of phenology and lifespan. In interspecific comparisons, we observed significant variation in total N uptake (*p* < 0.01), NUE (*p* < 0.01), and corresponding differences in biomass accumulation (*p* < 0.01; Supplementary Table S3). These results are well illustrated by contrasting *E. crus-galli* and *A. powellii*, which had considerable differences in N uptake despite being harvested on the same date; and are also illustrated by contrasting *E. tef* and *S.* × *drummondii*, which had a twofold difference in N uptake despite their similar phenology (Supplementary Figure S2). This variation may be partly attributed to seed size. For instance, the extremely small-seeded *E. tef* had relatively low biomass and N uptake residuals. Yet this was not a consistent trend as *S.* × *drummondii* and *H. annuus* had comparable biomass accumulation and N uptake despite large differences in seed size (Supplementary Figure S2). In contrast to interspecific comparisons, we did not observe significant variation in N uptake among maize inbred lines (*p* = 0.24; Supplementary Table S3). Rather, differences in biomass accumulation between maize genotypes (*p* < 0.01) were associated with differences in NUE (*p* < 0.01; Supplementary Table S2). As expected, nitrogen fertilizer significantly improved plant growth, N uptake and also lowered plant NUE (*p* < 0.05; Supplementary Figure S2 and Supplementary Table S2).

### Extracellular Enzyme Activity in the Rhizosphere

**FIGURE 1:**
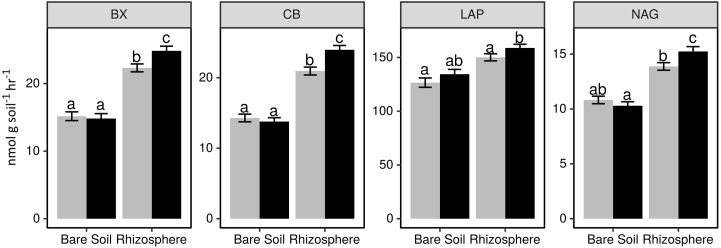
Potential activity of extracellular beta-xylosidase (BX), cellobiohydrolase (CB), leucine aminopeptidase (LAP), and *N*-acetyl-glucosaminidase (NAG) in bare soil and rhizosphere samples from plots receiving 0 kg N ha^-1^ (gray bars) and 95 kg N ha^-1^ (black bars). Letters indicate a significant difference between treatments (Tukey HSD *p* < 0.05). Note that scale of *y*-axis differs among plots.

We observed a significant stimulation of hydrolytic enzyme activity in the rhizosphere (**Figure 1** and Supplementary Table S4). This rhizosphere effect was modulated by nitrogen fertilizer addition, whereby CB, BX, and NAG activity increased in the rhizosphere of plants receiving fertilizer, but not in fertilized bare soil plots. In contrast, there was a trend toward increased LAP activity in both bare soil and rhizosphere samples that received fertilizer (**Figure 1** and Supplementary Table S4). The fertilizer effect exhibited a positive correlation between inorganic N concentration and potential enzyme activity in the rhizosphere (*p* < 0.05; Supplementary Table S5). Enzyme activity in the rhizosphere also differed between plant genotypes (*p* < 0.05; Supplementary Table S6), however, this result was only observed when comparing plants with different sampling dates. These differences between dates were not associated with a trend toward increasing or decreasing activity over the growing season (data not shown).

### Rhizosphere Effect on Bacterial Community Composition

**FIGURE 2:**
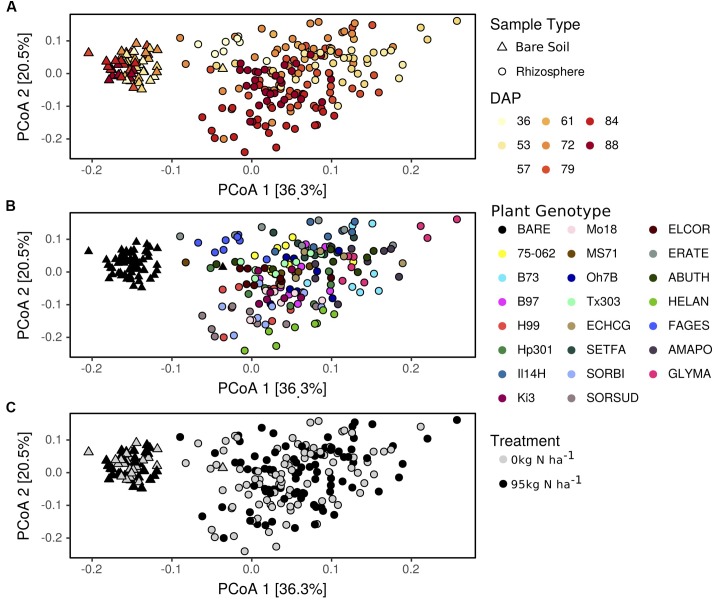
Bacterial community composition varies between rhizosphere and bulk soil and with respect to days after planting **(A)**, plant genotype **(B)**, and nitrogen fertilization **(C)**. Changes in bacterial community composition are visualized as a principal coordinate analysis (PCoA) of weighted-UniFrac distances between samples. Genotype codes represent maize inbred lines and species: *E. crus-galli* (ECHCG), *S. faberi* (SETFA), *S. bicolor* (SORBI), *S.* x *drummondii* (SORSUD), *E. coracana* (ELCOR), *E. tef* (ERATE), *A. theophrasti* (ABUTH), *H. annuus* (HELAN), *F. esculentum* (FAGES), *A. powellii* (AMAPO), and *G. max* (GLYMA).

**FIGURE 3:**
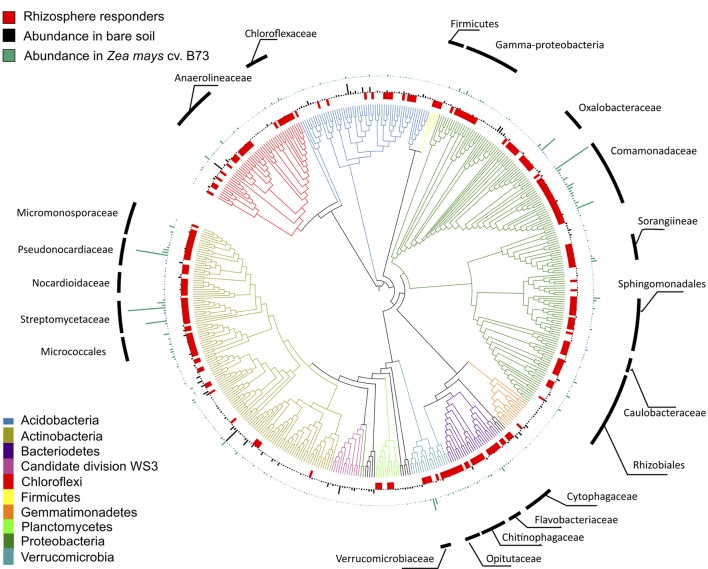
Phylogeny of 500 most abundant bacterial taxa in common garden experiment. From inner circle outward: red tiles indicate taxa significantly enriched in the rhizosphere of at least one genotype compared to bare soil controls collected on the same date (DESeq2: log_2_-fold change > 0.5; adjusted *p* < 0.05), black bars indicate mean relative abundance of OTUs in bare soil samples, green bars indicate mean relative abundance of OTUs in rhizosphere samples from *Zea mays* cv. B73, chosen to represent the rhizosphere effect in general. Tree created using the interactive tree of life (iTOL) web server.

**FIGURE 4:**
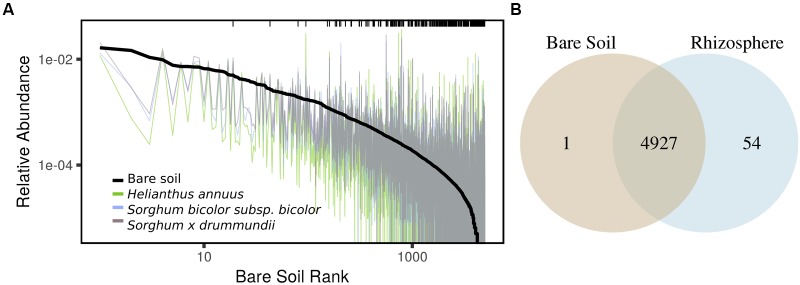
**(A)** Variation in OTU mean relative abundance from bare soil to rhizosphere of three representative species sampled on a single date. Black line indicates rank abundance in bare soil and colored traces indicate shifts in OTU relative abundance in rhizosphere samples. Black ticks indicate positive rhizosphere responders identified on any sampling date (DESeq2: log_2_-fold change > 0.5; adjusted *p* < 0.05), highlighting the enrichment in the rhizosphere of taxa at low abundance in bulk soil. **(B)** OTUs detected in bare soil and rhizosphere samples in full dataset.

In addition to shifts in enzyme activity, we observed a strong differentiation of BCC between bare soil and rhizosphere sample types (**Figure 2**). In a PERMANOVA of weighted-UniFrac distance, sample type was the greatest source of variation (**Table 1** and **Figures 2A–C**). Of the 4982 OTUs, 1502 were significantly enriched in the rhizosphere of at least one plant genotype compared with bare soils (**Figure 3**). Many of the rhizosphere responsive OTUs were at low abundance in bulk soil, but obtained high abundance in the rhizosphere (**Figure 4A**) resulting in a dramatic shift in community composition. These rhizosphere responsive taxa included 54 OTUs that were present in rhizosphere samples but not detected in bulk soil (**Figure 4B**), which could result from rhizosphere enrichment of extremely rare taxa or vertical transmission of root endophytes.

**Table 1:** Permutational multiple analysis of variance testing main effects of sample type (rhizosphere vs. bare soil), days after planting (DAP), plant genotype or species identity (genotype), and nitrogen fertilization treatment (0, 95 kg N ha^-1^) on bacterial community beta-diversity (weighted-UniFrac).

Factor	SS	DF	*F*	*R*^2^	*p^∗^*
**Full dataset**					
Sample type	2.24	1	141.49	0.25	**<0.01**
DAP	1.47	7	13.20	0.17	**<0.01**
Genotype	1.80	21	5.39	0.20	**<0.01**
N treatment	0.04	1	2.65	0.005	**0.03**
Residuals	3.28	207		0.40	
**Rhizosphere**					
DAP	1.97	7	14.87	0.33	**<0.01**
Genotype	1.16	14	4.37	0.19	**<0.01**
N treatment	0.06	1	2.92	0.01	**<0.01**
Residuals	2.85	151		0.47	
**Bare soil**					
DAP	0.13	1	2.61	0.25	**<0.01**
N treatment	0.009	1	1.19	0.02	0.19
Residuals	0.41	61		0.73	

^∗^*P-values based on 999 permutations. *P*-values < 0.05 highlighted in bold*.

Taxa enriched in the rhizosphere belonged to diverse phyla (**Figure 3** and Supplementary Table S7), but clustered within several groups. The *Proteobacteria* and *Actinobacteria* accounted for 23.6% and 16% of median relative abundance in rhizosphere samples and taxa from the *Bacteroidetes*, *Chloroflexi*, *Verrucomicrobia*, and *Firmicutes* also comprised a substantial fraction of the rhizosphere community (**Figure 3** and Supplementary Table S7). Within these phyla, some families showed a strong rhizosphere preference. The branching depth of clades sharing a phenotype can indicate the degree of phylogenetic conservation of that trait (Martiny et al., 2013). In our dataset, clades with more than 90% of OTUs displaying a rhizosphere response had a deeper average branching depth than expected under a permuted null model (Tau *D* = 0.02, *p* = 0.04; consenTRAIT), indicating phylogenetic conservation of the rhizosphere response within these families. These included families within the *Proteobacteria* (*Comamonadaceae*, *Oxalobacteraceae, Caulobacteraceae*, and *Sphingomonadaceae*), *Actinobacteria* (*Streptomycetaceae*), *Firmicutes* (*Bacillacae* and *Paenibacillaceae*), *Bacteroidetes* (*Flavobacteriaceae* and *Chitinophagaceae*), *Verrucomicrobia* (*Opitutaceae*), *and Chloroflexi* (*Chloroflexaceae*).

Nitrogen fertilization had a statistically significant, but very small, influence on BCC, accounting for <1% of the variation in the PERMANOVA of rhizosphere samples, which is not easily perceptible in the ordination (**Table 1** and **Figure 2C**). Nitrogen fertilization led to the enrichment of 118 OTUs and decline of 45 OTUs in relative abundance within plant rhizospheres (Supplementary Figure S3; log_2_-fold change ≠ 0, *p* < 0.05). Many of the OTUs responding positively to N fertilization were from the *Proteobacteria, Actinobacteria*, and *Bacteroidetes*, though these phyla also had representatives that decreased in abundance in response to N fertilization (Supplementary Figure S3). Several *Nitrospiraceae* and a single *Nitrosomonadaceae* increased in abundance, while several other *Nitrosomonadaceae* decreased in abundance in rhizosphere samples from fertilized plots (Supplementary Figure S3). In comparison, relatively few OTUs were correlated with inorganic N concentration in the rhizosphere, which suggests that the effects of fertilization on BCC in the rhizosphere are indirect, being driven less by the availability of mineral N and more by changes in plant growth and physiology, which occur in response to fertilizer. This result is not unexpected since plants were sampled at anthesis and not immediately after fertilization. In addition, fertilization did not have a significant impact on BCC in bare soil (**Table 1** and **Figure 2C**), further emphasizing the role of plants in mediating the observed response of BCC to fertilization.

### Plant Genotype Shapes Rhizosphere Bacterial Community Composition

Plant genotype and flowering time strongly shaped rhizosphere BCC as indicated by the PERMANOVA (**Table 1**). Variation between genotypes sampled on different dates accounted for 33% of the variation in rhizosphere BCC (**Table 1** and **Figure 2A**). Sampling date also explained a significant portion of variance in bare soil samples (**Table 1** and **Figure 2A**), however, both the total variance and the proportion of variance explained by sampling date were greater in rhizosphere samples than in bulk soils (**Figure 2**) and rhizosphere BCC varied dramatically from bulk soils (**Figure 2**). Thus, the variation between genotypes sampled on different dates cannot be explained by temporal variation in bulk soils and must be due to either plant genotype effects or unmeasured interactions between plant rhizosphere effects and time.

When added sequentially to the PERMANOVA to control for sampling date, plant genotype accounted for 19% of the variation in rhizosphere samples (**Table 1**). In this model, some plant genotype effects are attributed to sampling date, which provides a conservative estimate of plant genotype influence on the rhizosphere community. It is possible to eliminate sampling date effects on those dates when multiple genotypes were sampled. When assessed within a sampling date, plant genotype explained between 13% (*p* = 0.09) and 43% (*p* < 0.01) of variance in rhizosphere BCC (Supplementary Table S8). Genotype was also an important predictor of intraspecific variation in rhizosphere BCC. When evaluating just the maize inbred lines, sampling date accounted for 10% of the variance [*F*_(2,76)_ = 6.44; *p* < 0.01] and plant genotype explained an additional 26% [*F*_(8,76)_ = 3.95; *p* < 0.01] of the variance in rhizosphere BCC.

### Plant Phylogeny and Growth Characteristics Explain Differences in Rhizosphere Bacterial Community Composition

**FIGURE 5:**
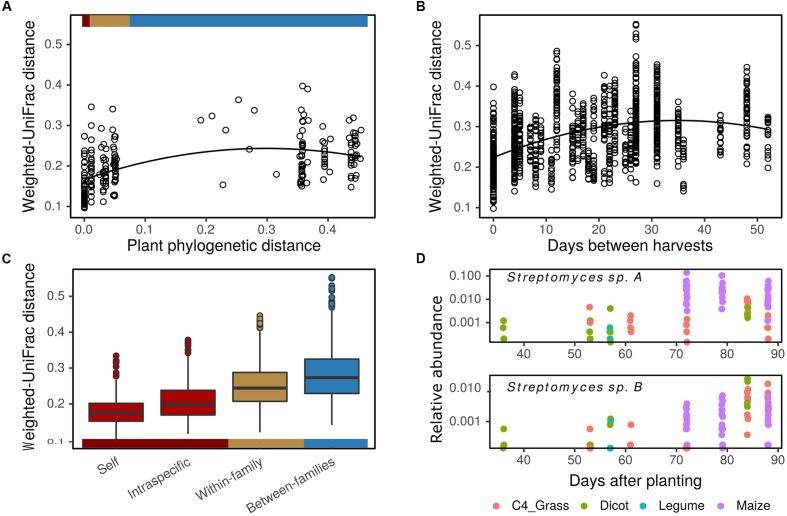
Plant phylogeny and flowering time influence rhizosphere bacterial community composition. Rhizosphere bacterial community beta-diversity (weighted-UniFrac distance) is positively correlated with plant host phylogenetic distance **(A)**, days between sampling **(B)**, and increases with plant host taxonomic rank **(C)**. Colored bars indicate correspondence of plant phylogenetic distances and taxonomic rank between **(A,C)**. Influence of time and plant phylogeny on bacterial taxa abundance is illustrated with two *Streptomyces* OTUs **(D)**. OTUs increase in abundance over time as a result of selective enrichment in maize (top panel) or enrichment over time independent of plant phylogeny (bottom panel) (DESeq2: log_2_-fold change per day = 0.08 ± 0.01 and 0.07 ± 0.01 in top and bottom panels, respectively; *p* < 0.05). Weighted-UniFrac distances calculated on mean genotype OTU abundances **(A)** and plot level OTU abundances **(B,C)**.

**FIGURE 6:**
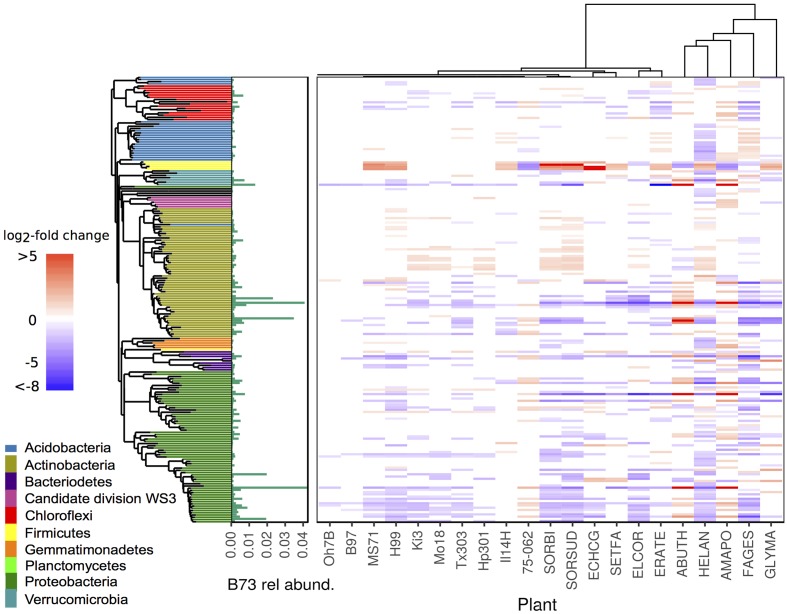
Differential abundance between rhizosphere of *Zea mays* cv. B73 and other maize genotypes and annual species. Tiles arranged by plant phylogeny (top tree) and bacterial phylogeny (left tree). Colored tiles indicate significant difference between listed genotype and reference B73 (DESeq2: log_2_-fold change ≠ 0, adjusted *p* < 0.05), color and intensity indicate direction and magnitude of log_2_-fold change. Green bars represent mean relative abundance in rhizosphere samples of B73. Genotype codes represent maize inbred lines and species: *E. crus-galli* (ECHCG), *S. faberi* (SETFA), *S. bicolor* (SORBI), *S.* x *drummondii* (SORSUD), *E. coracana* (ELCOR), *E. tef* (ERATE), *A. theophrasti* (ABUTH), *H. annuus* (HELAN), *F. esculentum* (FAGES), *A. powellii* (AMAPO), and *G. max* (GLYMA).

To evaluate the influence of plant phylogeny on rhizosphere BCC we constructed an MLPE model using a weighted-UniFrac distance matrix of average OTU relative abundance for each plant genotype. Beta-diversity increased in relation to plant phylogenetic distance through the taxonomic rank of family (**Figures 5A,C**), explaining 8% of the variation between plant rhizosphere communities (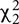 = 19.17, *p* < 0.01). This effect was robust when controlling for variation in BCC attributed to pairwise sampling date comparisons (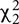 = 13.50, *p* < 0.01) and when intraspecific comparisons were removed from the dataset (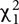 = 7.93, *p* < 0.01). Beta-diversity appeared to plateau beyond the rank of family, such as when comparing between grasses and dicots, suggesting that plants with similar characteristics had similar effects on rhizosphere BCC. The phylogenetic signal was evident in overall beta-diversity and at the level of individual OTUs (**Figure 6**). Non-maize species had more differentially abundant OTUs than maize genotypes when compared to maize reference line B73 (DESeq2: log2-fold change ≠ 0; BH adjusted *p* < 0.05). In addition, changes in relative abundance for the differentially abundant OTUs were greater for non-maize species than for other maize inbred lines when compared to maize reference line B73 (**Figure 6** and Supplementary Figure S4).

Flowering time, which explained most variation in plant growth and N economy (Supplementary Figure S1), was also the best continuous predictor of beta-diversity in plot level data. Rhizosphere beta-diversity increased with time between sampling dates in a polynomial fashion (**Figure 5B**), accounting for approximately 28% of the variation in rhizosphere BCC (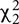 = 77.63, *p* < 0.01). Beta-diversity also increased with differences in plant NUE and seed size (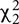 = 37.08, *p* < 0.01), but these factors only explained an additional 2% of the variation in rhizosphere BCC. The effect of sampling date could have multiple drivers including differences in physiology of plants with different lifespan, seasonal variation in soil characteristics, and temporal autocorrelation between sampling dates. There was also a relationship between flowering time and plant phylogenetic distance (*r* = 0.56, *p* < 0.01). Maize and many of the C4 grasses flowered later in the season while four of five dicots flowered early in the season. As a result, temporal variation in the relative abundance of OTUs will be driven both by plant species specific rhizosphere effects and by temporal variation in background soils. We highlight two *Streptomyces* OTUs to illustrate these patterns (**Figure 5D**). One is responsive to maize and related crop plants in the subfamily Andropogoneae (*S. bicolor* and *Sorghum* × *drummondii*). This maize responsive OTU is found in highest relative abundance during anthesis for maize and *Sorghum*, but it remains in low abundance in the rhizospheres of other plants sampled on these same dates (**Figure 5D**, top panel). In contrast, a second OTU from *Streptomyces* increases in abundance less specifically, responding to a range of plant genotypes including both C4 grasses and *H. annuus* (**Figure 5D**, bottom panel).

Intraspecific variation in rhizosphere BCC was not correlated with genetic distance (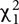 = 1.51, *p* = 0.22). Nor was beta-diversity correlated with variation in functional measures including flowering time, plant NUE or N uptake (*p* > 0.05).

### Changes in Bacterial Community Composition and Activity Associated with Plant Resource Acquisition and Use Strategies

**FIGURE 7:**
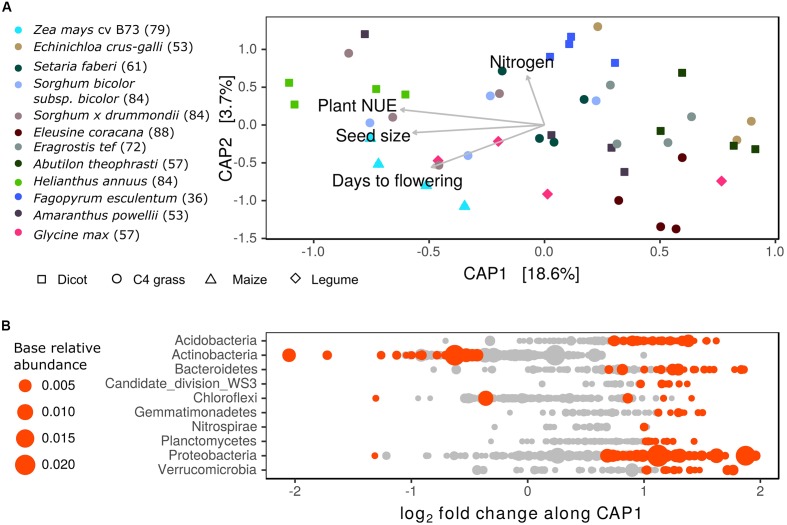
Relationship between plant life history strategy and bacterial community composition (BCC) in the rhizosphere. **(A)** Constrained analysis of principle coordinates (CAP) displaying variation in BCC explained by plant growth characteristics: days to flowering, seed size, plant nitrogen use efficiency (NUE) (g C g N^-1^) and nitrogen uptake (g N). Numbers in parentheses alongside species names in figure legend refer to sampling date as days after planting. Nitrogen use efficiency and N uptake are corrected for differences in sampling date by using residuals of model relating growth characteristics to date of harvest. **(B)** Estimates of log_2_-fold change in bacterial OTU abundance per unit shift in sample score on CAP1. Points colored in red are OTUs significantly correlated with CAP1 (DESeq2: log_2_-fold change ≠ 0; adjusted *p* < 0.05), gray points indicate OTUs not significantly correlated with CAP1 (*p* > 0.05). Point size proportional to relative abundance.

To further explore the role of plant growth characteristics in shaping variation in rhizosphere BCC between species, a principal coordinate ordination of weighted-UniFrac distances was constrained by explanatory growth characteristics including: days to flowering, seed size, N uptake and NUE. The constrained ordination explained roughly 22% of the variation in BCC (*p* < 0.01) (**Figure 7A** and Supplementary Table S9). The primary axis was negatively correlated with traits defining a resource intensive life history: longer lifespan, larger seed size and higher NUE. Notably, grasses and dicots were intermixed along this axis, with *H. annuus*, maize, and *Sorghum* occupying one end of the spectrum while *E. crus-galli* and *A. theophrasti* occupied the opposite end. Additionally, flowering time was not the sole driver of differentiation (Supplementary Table S9). Instead, long-lived but low NUE plants such as *E. coracana* and *E. tef* grouped with early season *E. crus-galli* to the exclusion of late season but high NUE plants such as maize and *Sorghum*. This axis captured marked compositional changes in rhizosphere BCC, characterized by the enrichment of many *Actinobacteria* OTUs in association with longer season and higher NUE plants (**Figure 7B**). The second CAP axis represented variation in total plant N uptake independent of flowering time and explained a small portion (3.7%) of variance in rhizosphere BCC. This axis was correlated with several *Bacillus* OTUs as well as a few *Acidobacteria* and *Cyanobacteria*. Neither axis separated the legume, *G. max*, from the other species, possibly indicating the plant’s life history traits were more important to its placement on this axis than its ability to fix nitrogen.

The correspondence of rhizosphere BCC with plant growth characteristics coincided with shifts in enzyme activity in the rhizosphere. The potential activity of BX, LAP, and NAG were negatively correlated with sample scores on the primary CAP axis, while the secondary axis was positively correlated with the potential activity of BX and CB (**Table 2**). This finding links plant growth characteristics to variation in BCC and enzyme activity such that plants with resource intensive life history traits had higher enzyme activity and differences in BCC compared to plants with less resource intensive life history traits.

**Table 2:** Correlations between principle coordinates that explain plant life history and bacterial community composition relationships and potential activity of extracellular beta-xylosidase (BX), cellobiohydrolase (CB), leucine aminopeptidase (LAP) and *N*-acetyl-glucosaminidase (NAG) and inorganic N concentration in the rhizosphere.

	Pearson correlation coefficients				
	BX	CB	LAP	NAG	Inorganic N
CAP1^∗^	**-0.38**	**-**0.26	**-0.55**	**-0.29**	**0.35**
CAP2	**0.51**	**0.53**	0.10	**-**0.14	**-**0.11

^∗^*Constrained analysis of principle coordinates (CAP) from **Figure 7**. Correlations significant at *p* < 0.05 highlighted in bold*.

## Discussion

In a common garden experiment, we investigated the sources and extent of plant variation in rhizosphere community composition and activity. We observed distinct changes in BCC and enzyme activity, reflecting the different C and N status between rhizosphere and bulk soil. Within this context, we show that rhizosphere BCC and enzyme activity is modulated by plant species and genotype and that this effect is related to plant phylogeny and life history strategy.

### Rhizosphere Effect on Bacterial Community Composition and Metabolism

Shifts in BCC and enzyme activity from bare to rhizosphere soils reflect the altered energy status of the rhizosphere environment. Consistent with other studies, rhizosphere samples were dominated by *Proteobacteria* and *Actinobacteria* (Bulgarelli et al., 2012, 2015; Peiffer et al., 2013), which include many bacterial species that grow rapidly in response to the availability of labile carbon substrates (Goldfarb et al., 2011). Additionally, many OTUs enriched in the rhizosphere were phylogenetically clustered and found at low abundance in bare soil, which suggests that rhizosphere competence requires traits that are evolutionarily conserved and which may not be adaptive in bulk soil (Barret et al., 2011; Ofek-Lalzar et al., 2014; Shi et al., 2015).

The potential activity of cellulose, hemi-cellulose, protein and chitin degrading enzymes was consistently greater in the rhizosphere compared to bare soil, which is consistent with studies showing a positive rhizosphere effect on enzyme activity, SOM decomposition, and N mineralization in the rhizosphere (Herman et al., 2006; Zhu et al., 2014). Controls on enzyme production in soil can include nutrient demand, target substrate availability, energetic constraints, and nutrient constraints (Sinsabaugh and Moorhead, 1994; Allison and Vitousek, 2005; Geisseler and Horwath, 2008). Accordingly, increased enzyme activity in the rhizosphere could reflect substrate flow from plant roots and release of C limitation. Nitrogen fertilizer further increased activity in the rhizosphere for all enzymes assayed, which was not observed in bare soil. These results could indicate that microbes experience greater N limitation in the rhizosphere, or that labile C from the plant roots is necessary to take advantage of the increased nutrient availability (Averill and Finzi, 2011).

A surprising result is that, while fertilizer addition increased plant growth and rhizosphere enzyme activity, its addition explained little variation in BCC relative to the effects of plant species and genotype. There are well documented effects of N fertilization on soil BCC (Ramirez et al., 2012; Leff et al., 2015) and evidence that N fertilization can shift rhizosphere BCC (Zancarini et al., 2012). Inorganic N fertilizer can influence BCC through a variety of mechanisms including: immediate direct responses to inorganic N availability (Verhamme et al., 2011), short term indirect responses caused by the effect of fertilizer on plant growth (Paterson et al., 2006), and long term indirect effects of fertilizer on soil properties such as pH (Hallin et al., 2009). Furthermore, these mechanisms may interact such that short-term effects vary depending on the fertilization history of the site. We propose two explanations for the minimal effect of N fertilization on BCC that we observed. First, temporal decoupling between fertilizer application and sampling may minimize detection of direct fertilization effects on BCC. Second, we propose that long-term use of mineral fertilizer at this site has minimized the responsiveness of BCC to short term fertilization effects. We note that those OTUs that increased in abundance in response to fertilizer were not tightly coupled with inorganic N concentration in soil. This result suggests that the fertilizer effects we did observe on BCC were mediated indirectly by plant response to fertilizer, such as increased root growth and exudation. This would explain why fertilization enhanced enzyme activity without causing substantial changes in rhizosphere BCC and why fertilizer had little effect on BCC and enzyme activity in bare soils.

### Plant Identity Shapes Rhizosphere Bacterial Community

Our finding that 13–43% of the variation in beta-diversity on a single sampling date could be attributed to plant species or genotype is consistent with previous reports of variation within a single field (Peiffer et al., 2013; Edwards et al., 2015), yet some studies have reported little or no plant identity effect on rhizosphere BCC (Bulgarelli et al., 2012; Wagner et al., 2016; Leff et al., 2017). To some degree these conflicting reports are expected. Genotype influences are less apparent in analyses where multiple fields or sample types increase total variance of the BCC (Peiffer et al., 2013; Bulgarelli et al., 2015; Edwards et al., 2015). Heritable plant phenotypes that influence rhizosphere communities may also be most influential during specific growth stages (İnceoğlu et al., 2010) or within a particular soil context. For example, Bell et al. (2014) observed that willow cultivars grown in contaminated soils selected distinct rhizosphere fungal communities, while those grown in non-contaminated soils did not. By situating our study in a single field it is implicit that the genotype effects we observed may not always emerge. However, reduced environmental variation allows a deeper look at the factors driving plant genotype and species variation in rhizosphere BCC. Here we investigated the strength of two plant factors—plant evolutionary history and variation in growth and N economy—in predicting variation in rhizosphere BCC.

### Plant Phylogeny Shapes Rhizosphere Bacterial Community

Plant evolutionary history explained a significant portion of variation in rhizosphere BCC. This adds to a growing body of studies detailing a link between host phylogeny and microbiome composition (Ley et al., 2008; Redford et al., 2010; Brucker and Bordenstein, 2012b). Similar to Bouffaud et al. (2014) our experimental design centered around maize and the Poaceae. Here we demonstrate that a phylogenetic signal is evident in the rhizosphere of field grown plants, whereby increasing phylogenetic distance leads to a more dissimilar bacterial community. This relationship has important implications. First, it suggests the phylogenetic conservation of plant traits that influence BCC. As discussed by Bouffaud et al. (2014), several traits that exhibit phylogenetic conservatism are likely to influence rhizosphere communities. For instance, root morphology displays a phylogenetic signal coincident with mycorrhizal association (Brundrett, 2002; Comas et al., 2014). In addition, secondary metabolite pathways, which may serve as signals in host–microbe communication, are often conserved at the family level (Wink, 2003). Furthermore, host immune responses directly influence the composition of the root microbiome and can be conserved phylogenetically (Brucker and Bordenstein, 2012b; Lebeis et al., 2015). A second implication is that the phylogenetic structure of plant communities would be expected to cause long-term changes in soil BCC (Barberán et al., 2015). This may serve as a mechanism underlying the positive relationship between plant phylogenetic diversity and ecosystem functions (Flynn et al., 2011). Introducing phylogenetic diversity to agricultural systems, either through rotations or intercropping, could therefore represent a management tool to influence rhizosphere and soil BCC and ultimately influence nutrient cycling in these soils (Berthrong et al., 2013).

### Plant Life History Strategy Shapes Rhizosphere Bacterial Community

Beta-diversity in rhizosphere BCC varied in response to plant flowering time, seed size, and life-span independent variation in NUE. These observations are consistent with the hypothesis that variation in plant growth and N economy influence rhizosphere microbiome composition. Within annual agricultural fields, where fitness is limited to ruderal plants with high growth rates, life history strategies are primarily differentiated by lifespan, which leads to differences in plant biomass accumulation, N demand, and NUE. These terms, along with seed size, reflect key dimensions of plant form and function (Moles and Westoby, 2006; Díaz et al., 2016) and could be linked with variation in rhizosphere BCC through multiple mechanisms.

Variation in N-uptake between plant species has strong impacts on nitrogen cycling dynamics in soil (Tilman and Wedin, 1991), and may contribute to variation in rhizosphere BCC between plant genotypes (Bell et al., 2015; Moreau et al., 2015; Pathan et al., 2015). In our study, extended N uptake of longer-lived plants may exacerbate N limitation within rhizosphere bacterial communities. It is possible that actinobacterial OTUs, enriched in long-lived and high NUE plants, such as *H. annuus*, maize and *Sorghum*, have adaptations to withstand N limitation in the rhizosphere. *Actinomycetes* produce a range of extracellular enzymes to degrade organic matter in soil (McCarthy and Williams, 1992). This could provide access to soil N pools in an otherwise N limited environment and underlie the increase in putative N-accessing enzymes observed in the rhizosphere of longer-lived, high NUE plants.

Alternately, plant traits correlated with lifespan and NUE may alter BCC. For example, plants classified as nitrogen competitive (high uptake) or conservative (high NUE) have been found to vary in the quantity and composition of their root exudates (Kaštovská et al., 2015; Guyonnet et al., 2017). In turn, species with higher rates of exudation supported increased microbial growth, turnover and high rates of N transformations in the rhizosphere (Blagodatskaya et al., 2014; Kaštovská et al., 2015). Thus, it is possible that shifts in rhizosphere C flows in long-lived high NUE plants alter the rhizosphere bacterial community. There is a rich literature connecting plant growth strategies to litter quality and subsequent impacts on nitrogen cycling in soil (Diaz et al., 2004; Hawkes et al., 2005; Orwin et al., 2010). Our findings suggest that plant life history strategy can also have direct impacts on rhizosphere BCC and activity.

By sampling at the onset of flowering, we captured a primary dimension of plant variation while limiting the effects of plant development on BCC. Since the phenology of these species was not synchronized, we cannot rule out that temporal shifts in edaphic factors contributed to our results. Nevertheless, if seasonal effects, rather than endogenous plant effects, are the source of changes in rhizosphere BCC observed here, they remain directly related to realized rhizosphere communities as they impact and interact with plants in the field. Furthermore, the grouping of long-lived, low NUE species with short-lived species in the constrained ordination supports the interpretation of a strong plant life history mediated effect on rhizosphere bacterial communities. Sequential sampling or staggered plantings to synchronize developmental stage (e.g., Wagner et al., 2016) in similar field experiments will be necessary to disentangle the interrelated effects of plant variation in growth, life history, and temporal variation.

Neither genetic relatedness nor growth and N economy successfully described intraspecific variation among maize lines, despite differences in rhizosphere BCC between genotypes. In this regard our results are consistent with previous work where genetic distance, plant height and plant size have not predicted intraspecific variation in rhizosphere BCC (Peiffer et al., 2013; Leff et al., 2017). In contrast, ecophysiological measures related to carbon and nitrogen acquisition did parse variation in rhizosphere BCC between *Medicago* genotypes (Zancarini et al., 2013). While it seems clear from our data that different plant species have different impacts on rhizosphere BCC, which are associated with differences in life history traits and rhizosphere function, it is less clear how intraspecific variation in plant traits influences microbiome composition and function.

## Conclusion

We demonstrate that both plant phylogeny and life history traits, including variation in lifespan, growth and N economy, explain significant variation in rhizosphere BCC and enzyme activity. These results suggest that differences in plant functional traits drive variation in BCC and impact resource acquisition from soil, which likely has both short and long-term consequences for soil BCC and N-cycling dynamics. Crop selection, cover cropping and crop rotation are key management interventions in below ground processes in agricultural systems. If the rhizosphere phenotypes observed in this study are repeatable in other fields, then incorporating phylogenetic and functional diversity into crop rotations may provide a mechanism to manipulate plant-microbe interactions over time. Fully understanding the implications of plant-induced shifts in the rhizosphere and soil microbiome will be critical in selecting plants and beneficial rotations for maximal agronomic benefit.

## Author Contributions

BE, LD, and DB designed the research. BE performed the research. BE and NY performed the sample and data analysis. BE, LD, and DB wrote the manuscript with assistance from NY.

## Conflict of Interest Statement

The authors declare that the research was conducted in the absence of any commercial or financial relationships that could be construed as a potential conflict of interest.

## Footnotes

1 https://github.com/nspope/corMLPE
