# Supplementary material for: Plant Phylogeny and Life History Shape Rhizosphere Bacterial Microbiome of Summer Annuals in an Agricultural Field
Source: Front Microbiol. 2017 Dec 11;8:2414. doi: 10.3389/fmicb.2017.02414 (PMC5732146; doi:10.3389/fmicb.2017.02414)
Supplement: Supplementary file 1 [file Table_1.pdf]

Table S1 Plant genotypes grown in common garden experiment, sample types collected and GenBank accession numbers for chloroplast sequences of species or congeneric representative used to construct plant phylogeny.

Maize Line	Species	Common Name	Days to flowering	Family	Sub-family	Rhizosphere Enzymes	Inorganic N	Bacterial community	matK /rbcL GenBank accession:	Germplasm source
75-062	*Zea mays ssp. mays*		72	Poaceae	Panicoideae/ Andropogoneae	■	■	■		Courtesy of Margaret Smith
B73	"		79	"	"	■	■	■	X86563.2/ X86563.2	Courtesy of Ed Buckler
B97	"		79	"	"	■	■	■		USDA GRIN : PI 564682
H99	"		72	"	"	■	■	■		USDA GRIN : PI 587129
Hp301	"		79	"	"	■	■	■		USDA GRIN : PI 587131
Il14H	"		72	"	"	■	■	■		USDA GRIN : Ames 27118
Ki3	"		88	"	"	■		■		USDA GRIN : Ames 27123
Mo18W	"		88	"	"	■		■		USDA GRIN : PI 550441
Ms71	"		72	"	"	■	■	■		USDA GRIN : PI 587137
Oh7B	"		79	"	"	■	■	■		USDA GRIN : Ames 19323
Tx303	"		88	"	"	■		■		USDA GRIN : Ames 19327

Table S1 cont. Plant genotypes grown in common garden experiment, sample types collected and GenBank accession numbers for chloroplast sequences of species or congeneric representative used to construct plant phylogeny.

Species Code	Species	Common Name	Days to Flowering	Family	Sub-Family	Enzymes	Inorganic N	Bacterial community	Chloroplast matK /rbcL GenBank accession:	Germplasm source
ERATE	*Eragrostis tef*	Teff (Tiffany)	72	Poaceae	Chloridoideae/ Eragrostideae	■	■	■	HE586095.1 / HE577859.1	Hancock Seed Co.
ELCOR	*Eleusine coracana*	Finger millet	88	Poaceae	Chloridoideae/ Cynodonteae	■		■	HQ180864.1/ HQ182427.1	USDA Grin: PI 427233
SORBI	*Sorghum bicolor ssp bicolor*	Grain sorghum	84	Poaceae	Panicoideae/ Andropogoneae	■		■	EF115542.1/ AM849341.1	King's Agri-Seed
SORSUD	*Sorghum x drummondii*	Sudangrass	84	Poaceae	Panicoideae/ Andropogoneae	■		■		Johnny's Selected Seeds
ECHCG	*Echinochloa crus-galli*	Barnyard Grass	53	Poaceae	Panicoideae/ Paniceae/ Boivinellinae	■	■	■	966202092/ AM887871.1	Aurora, NY
SETFA	*Setaria faberi*	Giant foxtail	61	Poaceae	Panicoideae/ Paniceae/ Cenchrinae	■	■	■	KF163774.1/ KF163540.1	Aurora, NY
ABUTH	*Abutilon theophrasti*	Velvetleaf	57	Malvaceae	Malvoideae	■	■	■	HQ696683.1/ HM849734.1	Aurora
HELAN	*Helianthus annuus*	Sunflower (Mammoth)	84	Asteraceae	Asteroideae	■		■	AY215805.1/ AF097517.1	Livingston Seed Co.
FAGES	*Fagopyrum esculentum*	Buckwheat	36	Polygonaceae	Polygonoideae	■	■	■	JF829981.1/ D86285.1	Lakeview Organic Grain variety not stated
AMAPO	*Amaranthus powellii*	Pigweed	53	Amaranthaceae	Amaranthoideae	■	■	■	169146456/ KJ773261.1	Aurora, NY
GLYMA	*Glycine max*	Soybean cv Sheyenne	57	Fabaceae	Faboideae	■	■	■	AF142700.1/ Z95552.1	Lakeview Organic Grain

Table S2 Inorganic N concentration in bare soil and rhizosphere samples at harvest.

		Inorganic N (mg kg soil^{-1})*	
Plant	DAP	0 kg N ha^{-1}	95 kg N ha^{-1}
Bare soil	36	9.62 (0.91)	14.48 (1.75)
Bare soil	53	9.82 (0.48)	30.01 (2.37)
Bare soil	57	10.92 (0.87)	27.44 (4.69)
Bare soil	61	12.92 (0.67)	22.29 (1.39)
Bare soil	72	11.47 (1.55)	19.38 (1.29)
Bare soil	79	10.79 (0.63)	19.23 (1.18)
Bare soil	84	--	--
Bare soil	88	--	--
F. esculentum	36	11.12 (1.11)	20.41 (5.36)
A. powellii	53	5.43 (0.41)	12.13 (2.05)
E. crus-galli	53	10.4 (4.02)	12.73 (6.32)[a]
A. theophrasti	57	13.55 (3.19)	26.13 (2.08)
G. max	57	11.26 (1.56)	19.42 (2.16)
S. faberi	61	6.24 (0.38)	6.67 (0.44)
75-062	72	5.88 (0.91)	8.1 (1.86)
E. tef	72	6.02 (1.05)	7.36 (2.1)
H99	72	--	--
Il14H	72	--	--
MS71	72	8.99 (2.15)	15.7 (3.52)
B73	79	--	--
B97	79	5.55 (0.39)	6.25 (0.55)
Hp301	79	--	--
Oh7B	79	6.31 (0.61)	7.48 (0.51)
H. annuus	84	--	--
S. bicolor	84	--	--
S. x drummondii	84	--	--
E. coracana	88	--	--
Ki3	88	--	--
Mo18	88	--	--
Tx303	88	--	--

* Values are mean (± s.e.m.)

-- Indicates inorganic N samples not collected

[a] One outlier (55.33 mg kg soil^{-1}) removed in table and all inorganic N analyses

Table S3 Analysis of variance testing main effects of plant genotype, nitrogen (N) treatment (0, 95 kg N ha^{-1}) and interaction on plant growth metrics*

	Interspecific Comparisons				Intraspecific comparisons			
	SS	DF	F	*p*	SS	DF	F	*p*
Biomass								
Genotype	6.56	11, 33	11.24	**<0.01**	1.30	10, 30	3.99	**<0.01**
N treatment	0.55	1, 36	10.42	**<0.01**	0.17	1, 33	5.30	**0.03**
Genotype x Treatment	0.89	11, 36	1.52	0.17	0.27	10, 33	0.85	0.58
N uptake								
Genotype	4.00	11, 33	5.29	**<0.01**	0.88	10, 30	1.38	0.24
N treatment	2.39	1, 36	33.99	**<0.01**	1.54	1, 33	24.22	**<0.01**
Genotype x Treatment	1.19	11, 36	1.57	0.15	0.42	10, 33	0.65	0.76
NUE								
Genotype	2.63	11, 33	33	**<0.01**	1.40	10, 30	10.67	**<0.01**
N treatment	0.64	1, 36	36	**<0.01**	0.66	1, 33	50.32	**<0.01**
Genotype x Treatment	0.36	11, 36	36	**0.03**	0.24	10, 33	1.83	0.09

*Growth metrics of plant biomass, nitrogen uptake and nitrogen use efficiency (NUE: g C g N^{-1}) are corrected for harvest date by using residuals from best fit-model of plant growth by days after planting (see figure S2). *P*-values < 0.05 are highlighted in bold.

Table S4 Analysis of variance testing main effects of sample type (rhizosphere vs bare soil), nitrogen (N) treatment (0, 95 kg N ha^{-1}) and interactions on potential activity of extracellular beta-xylosidase (BX), cellobiohydrolase (CB), leucine aminopeptidase (LAP) and N-acetyl-glucosaminidase (NAG).

		SS	DF	F	*p*
BX					
	Sample type	224.5	1, 49	15.06	**<0.01**
	N treatment	55.8	1, 146	3.74	**0.05**
	S x N	96.3	1, 146	6.46	**0.01**
CB					
	Sample type	279.9	1, 43	20.00	**<0.01**
	N treatment	73.7	1, 147	5.26	**0.02**
	S x N	145.9	1, 147	10.43	**<0.01**
LAP					
	Sample type	1958.8	1, 47	4.65	**0.04**
	N treatment	3248	1, 147	7.70	**<0.01**
	S x N	9.3	1, 147	0.02	0.88
NAG					
	Sample type	59.6	1, 448	9.25	**<0.01**
	N fertilization	8.3	1, 147	1.28	0.26
	S x N	41.9	1, 147	6.50	**0.01**

P-values < 0.05 are highlighted in bold.

Table S5 Analysis of covariance testing correlation between inorganic nitrogen (N) concentration in the rhizosphere and potential activity of extracellular beta-xylosidase (BX), cellobiohydrolase (CB), leucine aminopeptidase (LAP) and N-acetyl-glucosaminidase (NAG).

		SS	DF	F	*p*	R^2_m
BX						
	Inorganic N[a]	211	1, 77	10.45	**<0.01**	.10
CB						
	Inorganic N	225	1, 79	9.92	**<0.01**	.12
LAP						
	Inorganic N	6155	1, 69	19.70	**<0.01**	.11
NAG						
	Inorganic N	81	1, 77	12.96	**<0.01**	.14

* Marginal R^2 from a model testing inorganic N concentration as a fixed effect and plant genotype, rep and main plot as random effects. *P*-values < 0.05 are highlighted in bold.
[a]only samples for which inorganic N measured (Table S1) are included in analysis.

Table S6: Analysis of variance testing fixed effects of plant genotype and nitrogen (N) fertilization (0, 95 kg N ha^{-1}) on potential activity of extracellular beta-xylosidase (BX), cellobiohydrolase (CB), leucine aminopeptidase (LAP) and N-acetyl-glucosiminidase (NAG) in rhizosphere samples.

		SS	DF	F	*p*
BX					
	Genotype	1.00	10, 30	3.75	**<0.01**
	N treatment[a]	0.34	1, 32	12.68	**<0.01**
	P x N	0.33	10, 31	1.24	0.31
CB					
	Genotype	824	10, 29	4.04	**<0.01**
	N treatment	288	1, 32	14.12	**<0.01**
	P x N	190	10, 32	0.93	0.52
LAP					
	Genotype	20710	10, 30	6.02	**<0.01**
	N treatment	4327	1, 32	12.58	**<0.01**
	P x N	5481	10, 31	1.59	0.15
NAG					
	Genotype	0.98	10, 30	3.81	**<0.01**
	N treatment	0.99	1, 32	38.42	**<0.01**
	P x N	0.20	10, 32	0.78	0.64

[a] Only samples for which inorganic N measured (Table S1) are included in analysis. *P*-values < 0.05 highlighted in bold.

Table S7: Phylum statistics of rhizosphere bacterial community composition at OTU level and aggregated by phylum.

Phylum	# OTU responders*	OTU Relative Abundance			Phylum Relative Abundance		
		Min	Median	Max	Min	Median	Max
Proteobacteria	507	0	7.92E-06	1.55E-03	1.40E-01	2.36E-01	4.13E-01
Bacteroidetes	206	0	0.00E+00	8.05E-04	1.62E-02	4.07E-02	7.40E-02
Actinobacteria	174	0	5.53E-05	2.95E-03	1.00E-01	1.60E-01	3.19E-01
Chloroflexi	158	0	1.83E-05	7.83E-04	1.75E-02	4.47E-02	9.11E-02
Planctomycetes	144	0	0.00E+00	2.92E-04	5.08E-03	1.17E-02	3.62E-02
Verrucomicrobia	93	0	8.95E-06	9.69E-04	1.07E-02	3.03E-02	4.25E-02
Firmicutes	58	0	0.00E+00	2.79E-03	2.37E-03	1.30E-02	9.87E-02
Acidobacteria	56	0	3.89E-05	9.63E-04	9.35E-03	1.95E-02	5.13E-02
Gemmatimonadetes	38	0	1.17E-05	4.00E-04	1.53E-03	4.81E-03	1.17E-02
Cyanobacteria	24	0	0.00E+00	6.77E-04	7.58E-04	2.48E-03	1.25E-02
Armatimonadetes	21	0	0.00E+00	2.72E-04	1.27E-03	2.10E-03	3.68E-03
Fibrobacteres	8	0	0.00E+00	4.76E-04	2.21E-05	5.45E-04	3.52E-03
Spirochaetes	5	0	0.00E+00	1.11E-03	1.89E-04	5.24E-04	5.52E-03
Candidate_division_BRC1	3	0	0.00E+00	2.98E-04	2.32E-05	8.57E-05	8.63E-04
Elusimicrobia	3	0	0.00E+00	2.02E-04	1.26E-05	1.38E-04	4.71E-04
Chlorobi	2	0	5.79E-06	2.23E-04	2.53E-05	1.04E-04	3.17E-04
Nitrospirae	2	0	0.00E+00	4.63E-04	0.00E+00	1.27E-04	9.27E-04
TM6	2	0	0.00E+00	8.77E-05	0.00E+00	3.17E-05	1.73E-04
Candidate_division_OD1	1	0	0.00E+00	1.23E-04	0.00E+00	3.79E-05	1.23E-04
Deinococcus-Thermus	1	0	0.00E+00	3.59E-04	0.00E+00	4.31E-05	3.59E-04

* Rhizosphere responders are identified in DESeq2 in comparison of samples from each genotype with bare soil control (log_2-fold change > 0.5, BH adjusted $p < 0.05$). Metrics represent minimum, median and max relative abundance in in samples aggregated by genotype.

Table S8: Permutational multiple analysis of variance testing main effects of plant genotype and species identity and nitrogen (N) treatment (0, 95 kg N ha^{-1}) on rhizosphere bacterial community beta-diversity (weighted-UniFrac) on each sampling date.

Factor	SS	DF	F	R^2	p^*	Factor	SS	DF	F	R^2	p^*
53 DAP						79 DAP					
Genotype	0.05	1	1.92	0.13	0.09	Genotype	0.11	3	1.77	0.15	**<0.01**
Treatment	0.03	1	1.06	0.08	0.36	Treatment	0.04	1	0.06	0.06	**0.02**
Residuals	0.26	13		0.79		Residuals	0.54	27		0.78	
57 DAP						84 DAP					
Genotype	0.12	1	3.46	0.20	**<0.01**	Genotype	0.14	2	4.36	0.28	**<0.01**
Treatment	0.03	1	0.94	0.05	0.43	Treatment	0.03	1	2.01	0.07	0.07
Residuals	0.46	13		0.75		Residuals	0.33	20		0.65	
72 DAP						88 DAP					
Genotype	0.44	4	6.58	0.43	**<0.01**	Genotype	0.29	3	6.53	0.41	**<0.01**
Treatment	0.02	1	0.92	0.02	0.38	Treatment	0.02	1	1.56	0.03	0.14
Residuals	0.57	34		0.55		Residuals	0.40	27		0.56	

* *p*-values based on 999 permutations; *p*-values < 0.05 highlighted in bold. For reference, genotypes harvested on each date include 53: *E. crus-gali* and *A. powellii*; 57: *A. theophrast* and *G. max*; 72: *E. tef,* 75-062, H99, Ms71, and Il14H; 79: B73, B97, Hp301 and Oh7B; 84: *S. biocolor*, *S. x drummondii* and *H. annuus*; 88: Ki3, Mo18W, Tx303. Data from day 36 and 61 not shown as only one genotype was sampled on each date.

Table S9 Permutation tests for marginal effects (Type III) of terms in constrained analysis of principal coordinates relating plant growth characteristics to rhizosphere bacterial community composition.

Factor	DF	SS	F	*p**
Days after planting	1,41	0.13	4.71	**<0.01**
Seed size[a]	1,41	0.07	2.52	**0.02**
NUE[b]	1,41	0.11	3.73	**<0.01**
N uptake[c]	1,41	0.10	3.35	**<0.01**

* *P*-values based on 999 permutations

[a] Log-transformed

[b,c] Plant nitrogen use efficiency (NUE: g C g N^{-1}) and total nitrogen (N) uptake corrected for days after planting using residuals from fit line in Figure S1.
