# Supplementary material for: Plant Phylogeny and Life History Shape Rhizosphere Bacterial Microbiome of Summer Annuals in an Agricultural Field
Source: Front Microbiol. 2017 Dec 11;8:2414. doi: 10.3389/fmicb.2017.02414 (PMC5732146; doi:10.3389/fmicb.2017.02414)
Supplement: Supplementary file 2 [file Presentation_1.pdf]

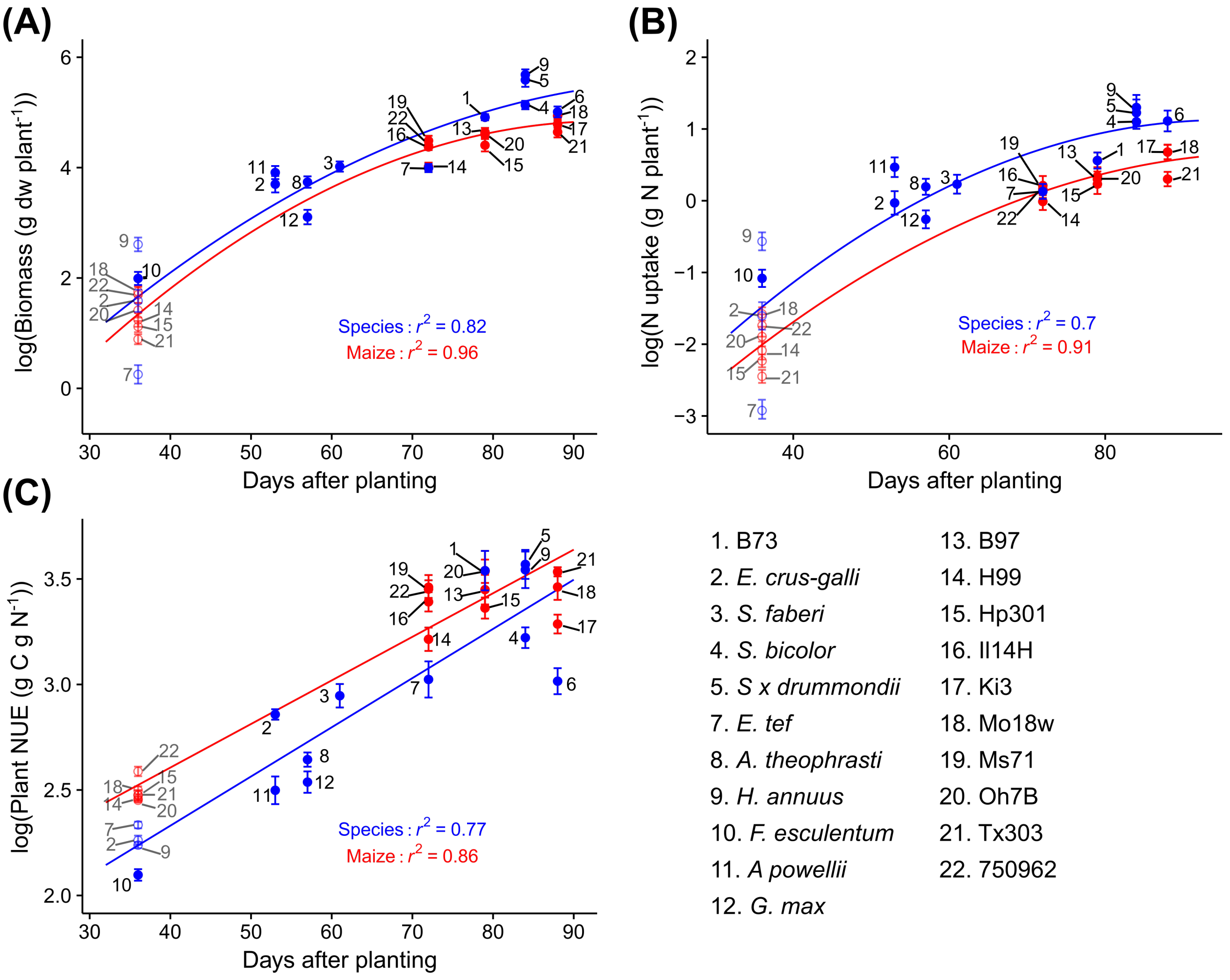

Figure S1: Days after planting vs log transformed plant biomass (A), nitrogen uptake (B) and plant nitrogen use efficiency (NUE) (C). Points are genotype mean ± 1 s.e.m. (n = 8). Line is fit against individual observations for annual species (blue) and maize inbred lines (red) separately using samples from both anthesis (closed circles) and, for a subsample of plants, vegetative stage (open circles).

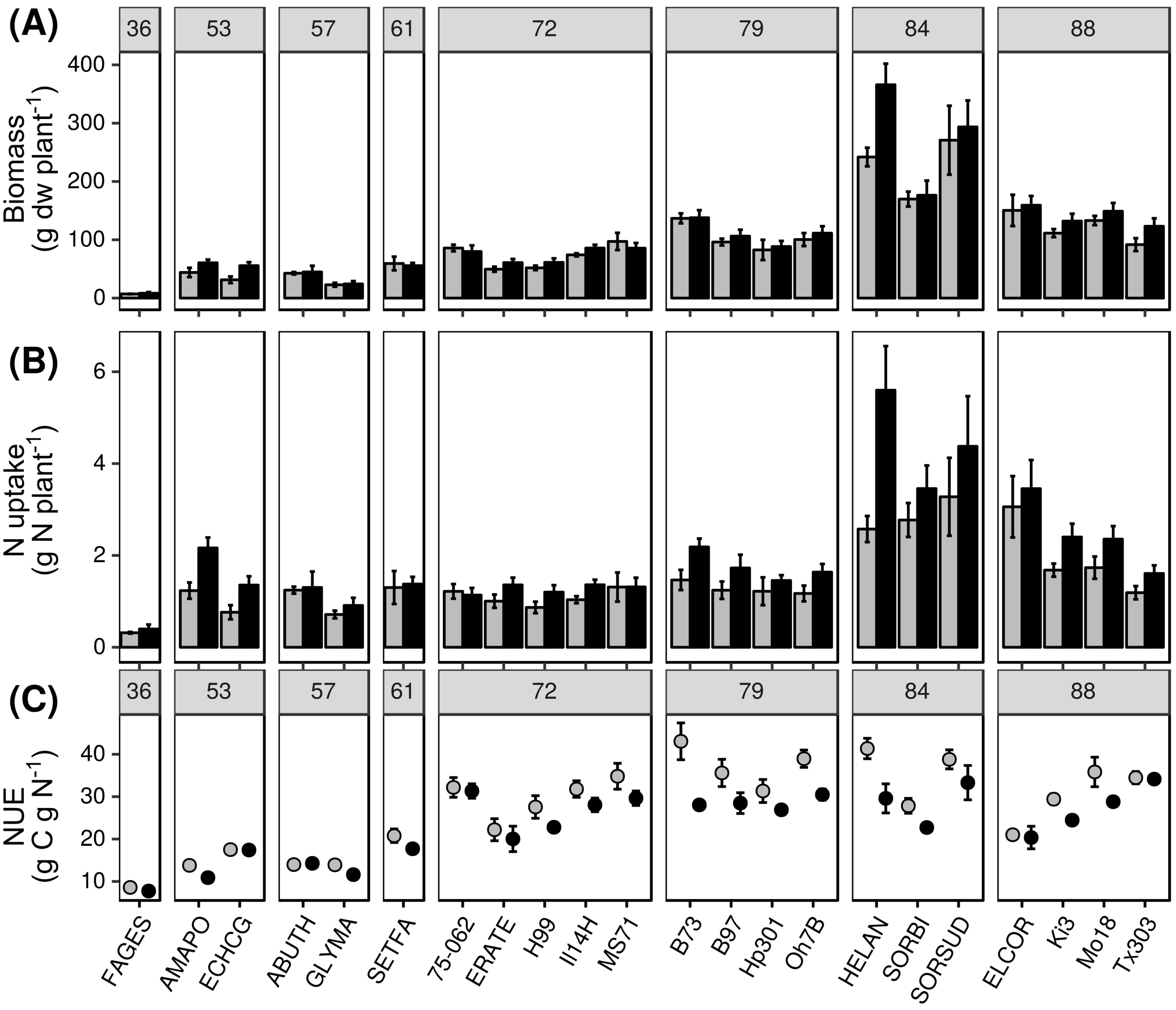

Figure S2: Cultivar and species variation in biomass accumulation, nitrogen (N) uptake, and nitrogen use efficiency (NUE) at anthesis under unfertilized (grey) and fertilized (black) conditions. Panels faceted by sampling date (days after planting). Bars are plant means ± 1 s.e.m. (n = 4). Species codes represent *E. crus-galli* (ECHCG), *E. tef* (ERATE), *S. faberi* (SETFA), *S. bicolor* (SORBI), *S. x drummondii* (SORSUD), *E. coracana* (ELCOR), *A. theophrasti* (ABUTH), *H. annuus* (HELAN), *F. esculentum* (FAGES), *A. powellii* (AMAPO), and *G. max* (GLYMA); remainder are maize inbred lines.

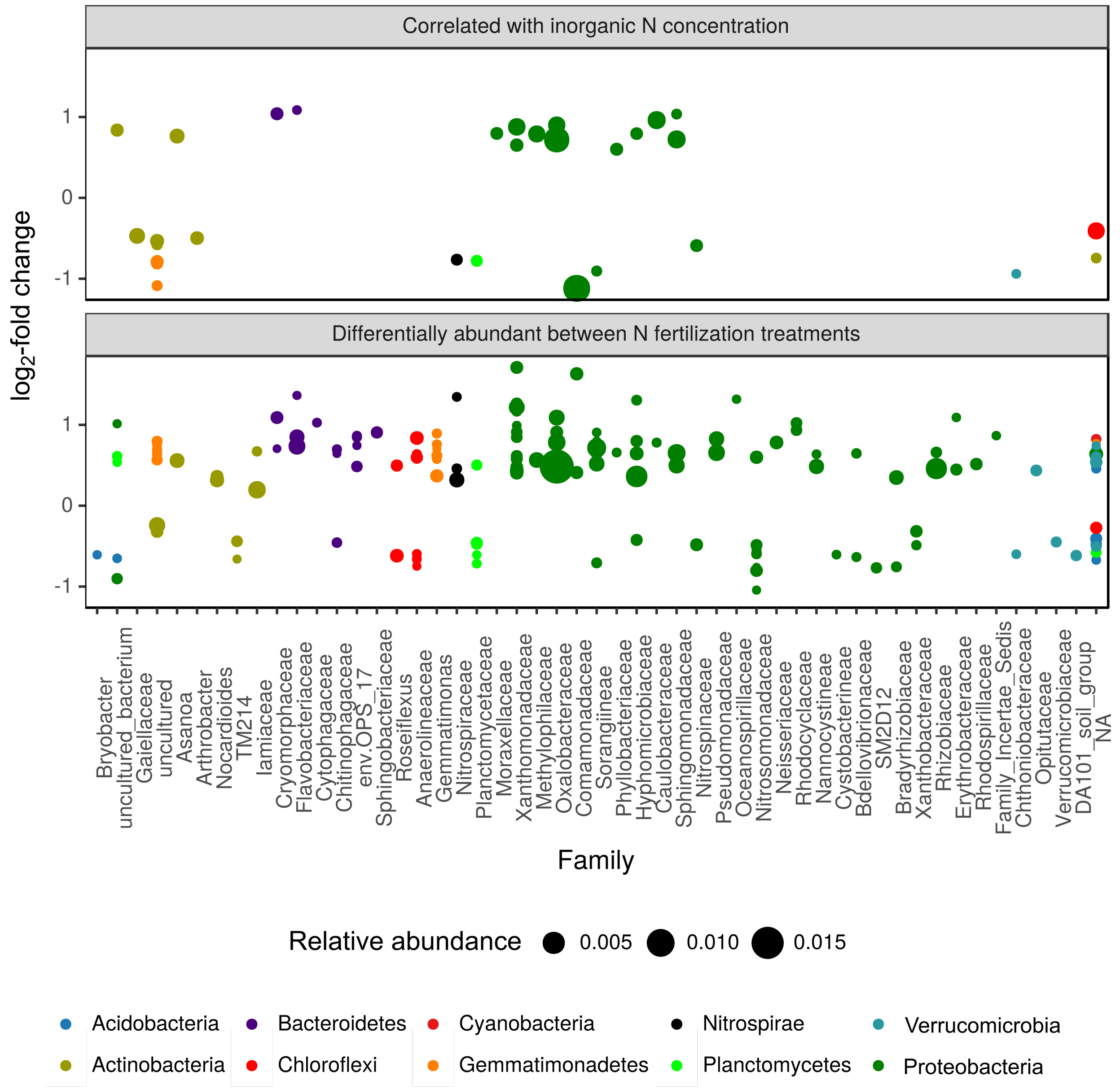

Figure S3: OTUs correlated with inorganic N concentration and differentially abundant between nitrogen fertilization treatments (0, 95 kg N ha^{-1}) in rhizosphere samples. Log_2-fold change in abundance with log-transformed inorganic N concentration (top panel; n = 84), and between fertilized and unfertilized plots (bottom panel; n = 174). Only OTUs with significant differential abundance shown (DESeq2: log_2-fold change ≠ 0, BH adjusted $p < 0.05$).

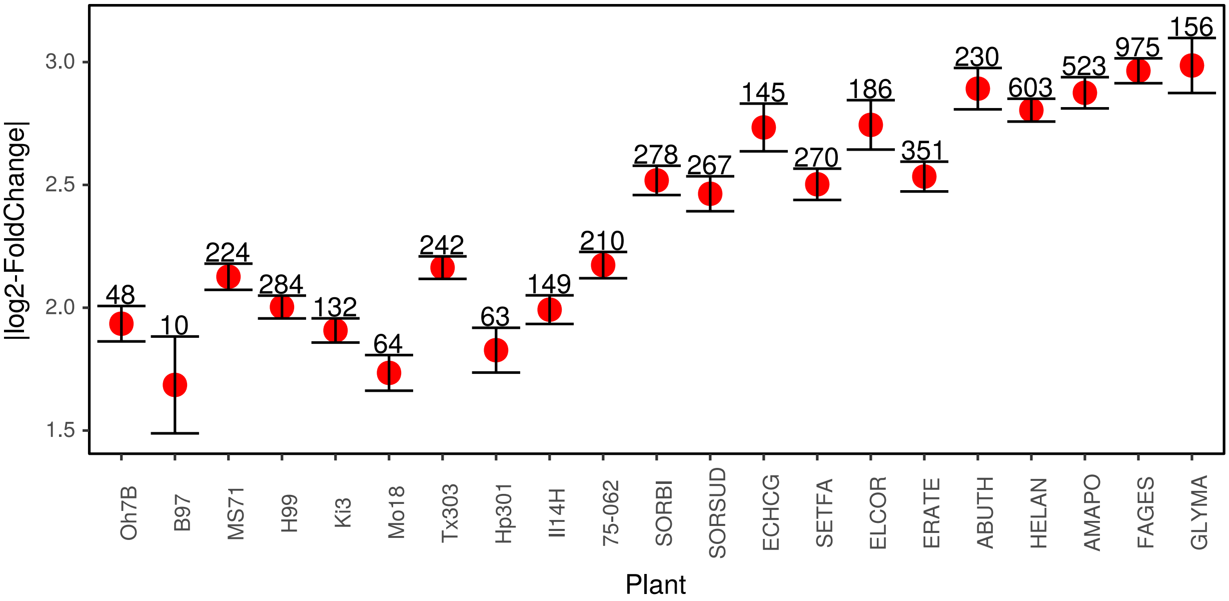

Figure S4: Number of OTUs differentially abundant between *Zea mays subsp mays* cv. B73 and each genotype and average magnitude of $\log_2$-fold change. Red dots indicate mean $\log_2$-fold change ± 1 s.e.m. of differentially abundant OTUs. Number of differentially abundant OTUs indicated above bars (n = 8, $\log_2$-fold change ≠ 0; BH adjusted $p < 0.05$).
